# Editorial: Methods and applications in neurorobotics

**DOI:** 10.3389/fnbot.2022.1111877

**Published:** 2022-12-15

**Authors:** Suparerk Janjarasjitt

**Affiliations:** Department of Electrical and Electronic Engineering, Ubon Ratchathani University, Ubon Ratchathani, Thailand

**Keywords:** robot, human machine interface, kinematics, electromyogram, electroencephalogram, deep learning, virtual reality

There are development and advancement on concepts and methods for applications in neurorobotics that are continually achieved and published. This Research Topic provides a mini collection of up-to-date technologies and methods which address challenges and tackle limitations in neurorobotics. Those technologies and methods collected in this Research Topic involve various applications in neurorobotics ranging from human machine interface, rehabilitation, and treatment.

Kinetic parameters and effects associated with the interaction between human and exoskeleton based on electromyogram (EMG) and muscular activities were evaluated in *Assessment methodology for human-exoskeleton interactions: Kinetic analysis based on muscle activation* (Fanti et al.). A new assessment protocol on human-exoskeleton interaction that requires a fewer number of instruments and leads to an easier process was proposed.

The influence of proprioceptive training on lower limb function in patients after a stroke was examined and presented in *Influence of proprioceptive training based on ankle-foot robot on improving lower limbs function in patients after a stroke* (Mao et al.). The experimental results suggest that the motor function and walking ability in patients after a stroke can be effectively improved from the proprioceptive training based on an ankle-foot robot.

In *ClueDepth Grasp: Leveraging positional clues of depth for completing depth of transparent objects* (Hong et al.), a deep learning technique was developed for improving a perception on transparent objects for machines and robots. The better accuracy and successful employment of a humanoid robot grasping were achieved. The ClearGrasp dataset that is a set of images of transparent objects is made publicly available at https://sites.google.com/view/~cleargrasp/data?authuser=0.

*Blind detection of circular image rotation angle based on Ensemble Transfer Regression and fused HOG* (Dong et al.) is another work that develops a deep neural networks model for improving a perception of machines and robots. The improved performance on accuracy of rotation angle estimation can be achieved using the ensemble transfer learning regression networks model.

A video game has been adopted as a part of treatment for children with attention-deficit hyperactivity disorder (ADHD). The brain activity of subjects during playing and interacting with a multitask game in virtual reality (VR) was focused in *The comparison of electroencephalography power and event related potential in success and failure during multitask game* (Sanuki et al.). A significant difference in EEG power associated with a theta band (4–7 Hz) was evidenced. This finding thus implies that the theta power of EEG acquired from the frontal area of the brain can be applied for predicting the success or failure of the Go trial.

## Author contributions

The author confirms being the sole contributor of this work and has approved it for publication.

